# Sequencing-based transcriptome analysis reveals diversification of immune response- and angiogenesis-related expression patterns of early-stage cervical carcinoma as compared with high-grade CIN

**DOI:** 10.3389/fimmu.2023.1215607

**Published:** 2023-09-04

**Authors:** Olga V. Kurmyshkina, Pavel V. Dobrynin, Pavel I. Kovchur, Tatyana O. Volkova

**Affiliations:** ^1^ Laboratory of Molecular Genetics of Innate Immunity, Institute of Medicine, Petrozavodsk State University, Petrozavodsk, Russia; ^2^ Human Genetics Laboratory, Vavilov Institute of General Genetics of Russian Academy of Sciences, Moscow, Russia; ^3^ Department of Hospital Surgery, Oncology, Urology, Institute of Medicine, Petrozavodsk State University, Petrozavodsk, Russia; ^4^ Hospital Admitting Department, The Republican Oncological Dispensary, Petrozavodsk, Russia; ^5^ Department of Biomedical Chemistry, Immunology and Laboratory Diagnostics, Institute of Medicine, Petrozavodsk State University, Petrozavodsk, Russia

**Keywords:** cervical cancer, transcriptome, tumor invasion, tumor microenvironment, preinvasive lesions, signaling pathways, angiogenesis, immune infiltration

## Abstract

**Background:**

Molecular diversity of virus-associated cervical cancer remains a relatively underexplored issue, and interrelations of immunologic and angiogenic features during the establishment of a particular landscape of the cervical cancer microenvironment are not well-characterized, especially for its earliest clinical stages, although this may provide insight into the mechanisms behind the differences in tumor aggressiveness, treatment responsiveness and prognosis. In this research, we were aimed at identifying transcriptomic landscapes of early-stage cervical carcinoma that differ substantially in their immune-related characteristics, patterns of signaling pathways and composition of the microenvironment in comparison with immediate precursor (intraepithelial) lesions.

**Methods:**

We performed the Illumina platform-based RNA sequencing using a panel of fresh tissue samples that included human papillomavirus-positive cervical intraepithelial neoplastic lesions (CIN), invasive squamous carcinoma of the cervix of FIGO IA1-IIB stages, and morphologically normal epithelium. The derived transcriptomic profiles were bioinformatically analyzed and compared by patterns of signaling pathway activation, distribution of tumor-infiltrating cell populations, and genomic regions involved.

**Result:**

According to hierarchical cluster analysis of the whole-transcriptome profiles, tissue samples were distributed between three groups, or gene expression patterns (the one comprising most pre-cancer cases and the other two encompassing mostly early-stage invasive cancer cases). Differentially expressed genes were retrieved in each intergroup pairwise comparison followed by Gene Ontology analysis. Gene set enrichment analysis of the two groups of tumor samples in comparison with the CIN group identified substantial differences in immunological and angiogenic properties between tumorous groups suggesting the development of different molecular phenotypes. Cell composition analysis confirmed the diverse changes in the abundancies of immune and non-immune populations and, accordingly, different impacts of the immune and stromal compartments on the tumor microenvironment in these two groups of tumors compared to CIN. Positional gene expression analysis demonstrated that the identified transcriptomic differences were linked to different chromosomal regions and co-localized with particular gene families implicated in immune regulation, inflammation, cell differentiation, and tumor invasion.

**Conclusions:**

Overall, detection of different transcriptomic patterns of invasive cervical carcinoma at its earliest stages supports the diverse impacts of immune response- and angiogenesis-related mechanisms on the onset of tumor invasion and progression. This may provide new options for broadening the applicability and increasing the efficiency of target anti-angiogenic and immune-based therapy of virus-associated cervical carcinoma.

## 1 Introduction

According to WHO data on global incidence and mortality rates of oncological diseases and the results of the worldwide statistical analysis of the Global Cancer Observatory database, cervical cancer (CeCa) is the fourth most common cancer in women and therefore continues to be a major health problem, being ranked in the top three cancers affecting middle-aged women in most countries (1). The reasons for this may arise from not only limitations in implementing preventive and screening measures or availability of medical care, but also constraints on new diagnostic and therapy approaches, which, in turn, may be brought about by insufficient understanding of pathogenesis mechanisms. Nonetheless, its virus-associated nature, with high-risk human papillomavirus (HPV) being the main etiological factor, and correspondingly deep involvement of innate and adaptive immunity boost an ever-increasing interest toward CeCa and its precursor lesions in recent years, especially in view of rapid advance in immunotherapy (2, 3). Current high-throughput analytical techniques provide researchers with an opportunity to construct tumor microenvironment (TME)-relevant classification systems, which can serve an essential basis for designing immunotherapeutic and/or antiangiogenic approaches (4, 5). Studies carried out in this field have uncovered the existence of relatively consistent immune landscapes within a given cancer type (6). It should be noted, however, that the problem of CeCa immunophenotype diversity in conjunction with other TME-related characteristics has only recently come under discussion. At the same time, such molecular phenotypes are a relatively well documented fact for many other cancer types.

On the whole, classification models proposed on the basis of expression profiles of immune-related genes and/or immune infiltration patterns are in accord with the concept of “hot/cold” tumors, or immune active/silent, or inflamed/noninflamed (7). In addition to “hot” and “cold”, an “altered” phenotype has also been recognized (8). By analyzing The Cancer Genome Atlas (TCGA) datasets some authors identified most cases of HPV(+) CeCa as the “hot” subtype (9, 10) evolving on the basis of chronically inflamed ТМЕ (11, 12). The defining features of this CeCa phenotype include high rates of immune infiltration, pro-inflammatory and Th1-dominant cytokine profiles, and a broad TCR repertoire believed to result from virus-induced genomic instability, high neoantigen load and a consequential immunogenicity (13). High immune activity in a tumor site intrinsically invokes inhibitory checkpoint mechanisms, which tumor cells take advantage of to combat immune response, thereby explaining why immune checkpoint inhibitor-based therapy is generally appreciated as most promising option (14). However, such categorization of CeCa molecular phenotypes presently seems insufficient to account for all the processes of active immunosuppression within the “hot” tumor type (partly accounting for low-to-moderate efficiency of immunotherapy in CeCa patients) and hence requiring a more in-depth investigation (15). Another challenging issue is the role of inflammation, a recognized promoter of oncogenesis and immune suppression (16, 17); at the same time, however, bioinformatics research on TCGA cases of cervical squamous cell carcinoma and endocervical adenocarcinoma (CESC-TCGA) has evidenced that higher patients’ survival rates correlated with higher immune scores (15). Recent studies also suggest that angiogenesis signature genes can help in stratifying CeCa molecular phenotypes and that there is a tight association between the angiogenic and immune expression profiles (18, 19).

Along with the problem of elaborating CeCa-specific immune- and angiogenesis-related molecular phenotypes, there are still many debatable questions regarding the earliest stages of CeCa progression (precancer lesions, pre- and microinvasive cancer stages), namely, how the diversity of CeCa “portraits” is formed and what are the putative determinants (20–22). Despite the widely studied mechanisms behind the action of HPV-oncogenes, there is still no clear understanding of how an immunologically latent (tolerogenic) infection and the ensuing benign hyperplasia transforms into a “hot”, heavily infiltrated and inflamed, neoplasia in certain cases and why the latter acquires further an immunosuppressive and exhausted phenotype (12). Some evidence indicates that determination of a CeCa immune “portrait” likely occurs at a pre-invasive stage, this being of potential value in point of developing treatment approaches to early stages (23). Furthermore, novel data from single-cell transcriptomics helped shed light on immunoregulatory activities of specific epithelial cell populations that can predetermine developmental trajectory of immune microenvironment of cervical high-grade neoplastic lesions towards immune activation resulting in co-enrichment of activated and exhausted effector T cells (24). At the same time, with the establishment of cervical microcarcinoma and its progression to invasive cancer, a marked increase in the diversity of co-expression meta-programs and immune heterogeneity, as well as global upregulation of interferon responses has been recently reported (25). Another source of complexity of CeCa immune landscape can be the recently described non-linear alterations of its specific components (such as regulatory and memory T cells) during progression from intraepithelial lesions to invasive cancer (26). However, since most CeCa molecular profiling studies addressed more advanced, metastatic, and recurrent disease stages, these data need to be additionally supported by enrollment of earlier clinical and preclinical stages. Addressing the above-described issues, we performed total RNA isolation and whole-transcriptome sequencing followed by bioinformatics analysis of a cervical tissue sample panel which included the earliest invasive stage and precancerous lesions obtained in our own hospital settings. We were aimed at showing detectability of distinct transcriptomic patterns that may reflect the formation of different immunophenotypes or ТМЕ-phenotypes upon transition to an invasive CeCa from its immediate intraepithelial precursor lesions.

## 2 Materials and methods

Tissue samples were obtained from patients with HPV(+) cervical intraepithelial neoplasia (CIN) of grades 1–3 (n=5; CIN3 comprised carcinoma *in situ*/CIS cases) and early-stage invasive squamous cell carcinoma of the cervix at FIGO stages IA-II (n=9; including 4 cases of microinvasive cancer with invasion of the stroma up to 3 mm in depth and 7 mm of extension, **Supplementary Figure S1**) during a colposcopy-directed biopsy or surgery (**Table 1**; **Supplementary Table S1**) (27). Patients underwent treatment in the Republican Oncological Dispensary; in each case, the diagnosis was histologically verified by histopathologists, who also inspected the prevalence of cancerous cells in malignant tissue with minimal inclusion of underlying loose connective tissue stroma to ensure the biopsy sampling accuracy. The presence of the high-risk carcinogenic HPV infection was confirmed in each case by the real-time PCR. No prior treatment was used before biopsy uptake. Two cases of morphologically normal cervical epithelium taken from healthy controls were also included. The research was approved by the Committee on Medical Ethics at the Institute of Medicine of Petrozavodsk State University and the Ministry of Healthcare of the Republic of Karelia (protocol No.5, Approval date: 1 Dec 2022), and was done in accordance with the Declaration of Helsinki and good clinical practice guidelines. The diagnosis was based on comprehensive physical examination, extended colposcopy findings, cytology and histopathology tests, in full compliance with the approved standards for the diagnosis and treatment of patients with gynecological malignancies. All women engaged in this this study were informed and gave voluntary written consent. Cervical tissue samples were placed in IntactRNA stabilization reagent at +4°C immediately after excision (during surgery). Total RNA was isolated using TriZOL reagent (Invitrogen, Carlsbad, CA, USA). The quality and quantity of isolated RNA were assessed based on 28S:18S rRNA ratio using Fragment Analyzer automated system (Advanced Analytical/Agilent, Santa Clara, CA, USA) and NanoDrop-2000 Spectrophotometer. Only samples that matched the quality control criteria were further processed for RNA sequencing (RNA-Seq).

**Table 1:** A panel of cervical tissue samples selected for RNA-Seq: designations and diagnosis.

Sample ID *	Degree/Stage
Norm1	morphologically normal cervical epithelium
Norm2	morphologically normal cervical epithelium
CIN_1	CIN3
CIN_2	CIN3 (carcinoma *in situ*, CIS)
CIN_3	CIN2/3 (high-grade squamous intraepithelial lesion)
CIN_4	CIN3 (CIS)
CIN_5	CIN1
CR_1	IA1
CR_2	IB1
CR_3	IA1
CR_4	IA1
CR_5	IB1
CR_6	IB2/IIA1
CR_7	IIB
CR_8	IA1
CR_9	IA2

*Each specimen, including normal epithelium, was obtained from a different patient. Pathological specimens were abbreviated as CIN_# or CR_#, where # is an ID number.

cDNA libraries were constructed using TruSeq stranded Ribo-Zero kit (Illumina, San Diego, CA, USA), reverse transcriptase SuperScript III (Invitrogen, Carlsbad, CA, USA), and AMPure XP Beads (Beckman, Brea, CA, USA). The adaptors-ligated purified fragments were loaded onto the flow cell using MiSeq v3 sequencing kit; 75 bp end-reads were generated on the MiSeq platform (Illumina, San Diego, CA, USA). Raw paired-end reads were filtered (sequence quality control was done with the FastQC tool); then, the filtered reads were mapped to the reference human genome (GRCh38/p13, NCBI) using STAR aligner to generate BAM-files and, further, calculate read counts. HTSeq package was used to assess the abundance of transcripts which was calculated by estimating the Counts Per Million reads mapped (CPM). Genes with minimum counts of 0.5 in at least one sample were considered for analysis. Pseudo count for log CPM was set to 4. EdgeR used as a method for counts data transformation (28). Ensembl gene IDs were converted to the corresponding NCBI gene IDs or gene symbol names. To estimate expression level of HPV-derived transcripts, the sequenced reads were mapped to high-risk HPV genome (https://pave.niaid.nih.gov) using HISAT2 aligner and counted using featureCount. The generated RNA-Seq dataset has been deposited in the National Center for Biotechnology Information Gene Expression Omnibus (GEO) with accession ID GSE223804.

The top 1000 most variable genes were selected for hierarchical clustering and heatmap construction. No normalization by gene or sample was used. Correlation was used as a distance function. Outliers beyond 4 SD were removed. K-means clustering was performed using 2000 most variable genes and 3 clusters considered the most optimal choice. Total transcriptional profiles were compared among the samples *via* principal component analysis (PCA) along the first two principal components. DESeq2 software was applied to study the differential gene expression (29). The genes with the base 2 logarithmic fold change value |logFC| larger than 1.0 and false discovery adjusted p-value (p-adj.) <0.1 were identified as Differentially Expressed Genes (DEGs). Gene ontology (GO) functional enrichment analysis were carried out on DEGs using Gene Ontology biological processes with an adjusted p-value of <0.05 and gene count of >2 considered as the thresholds; enrichment trees and networks were generated and visualized using ShinyGO web-tool (30). Pathway analysis for the patient groups’ comparisons was performed using the Generally Applicable Gene set Enrichment (GAGE) method (31) and the genes were annotated according to GO biological processes. The minimum and maximum gene set sizes were set to 15 and 2000 respectively, and the pathway significance cut-off was set to 0.2. The top 30 pathways were retrieved for each pairwise group comparison and visualized as hierarchical tree using ShinyGO. Identification of co-expression networks and sub-modules was performed using weighted gene co-expression network analysis, or WGCNA (32). The top 1000 most variable genes were included with a soft threshold of five and a minimum module size of 20 genes to construct a gene cluster tree and different modules. Functional enrichment analysis was performed for each module using GO biological processes and the resulting enriched pathway tables were exported. Cell-type enrichment analysis of bulk transcriptomes was performed using xCell deconvolution method (33), and enrichment scores of 64 immune and stroma cell types across samples were obtained. Position RElated Data Analysis (PREDA) was conducted to identify genomic regions significantly enriched with upregulated or downregulated genes (34).

## 3 Results

### 3.1 Comparative transcriptome and pathway analysis

**Figure 1:**
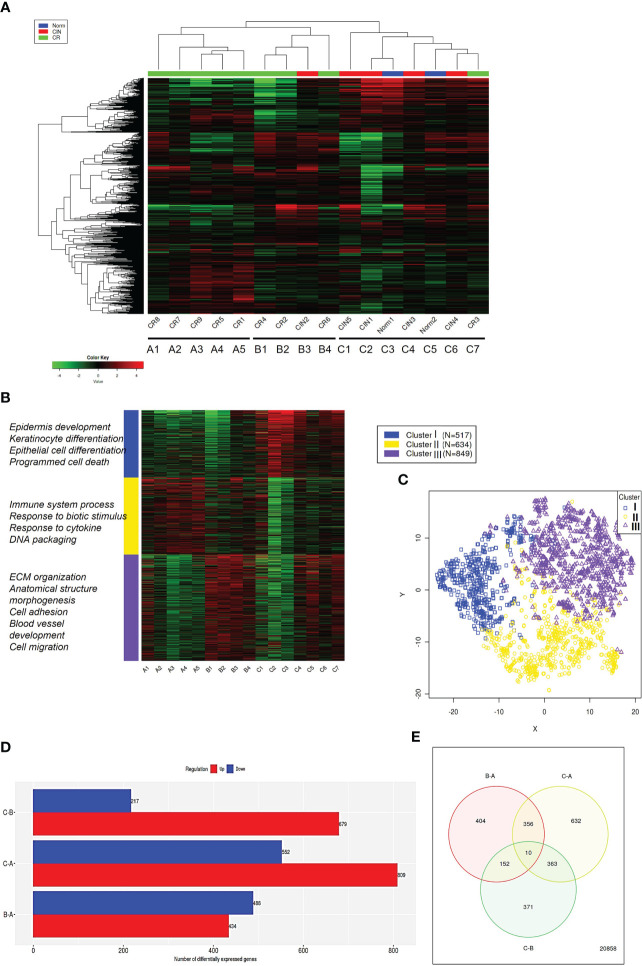
Comparative transcriptome analysis. **(A)** Hierarchical clustering performed on gene expression profiles of cervical lesions identified three main sample groups (‘A’, ‘B’, ‘C’) partially overlapping with pathological staging (CIN, CR). The heatmap was generated based on 1000 genes showing the largest expression variations across the samples (**Table S1** contains the list of top1000 genes identified and their mean normalized log-transformed counts used for heatmap construction). **(B)** K-Means cluster analysis performed on top 2000 most variable genes (k = 3). On the left are the top GO biological processes from each cluster (the list of top2000 genes and their normalized log-transformed counts used for heatmap construction are available on demand). **(C)** t-SNE plot of top2000 genes, color-coded by their associated cluster (X and Y axes are the first two t-SNE components). **(D)** Differential gene expression between ‘A’, ‘B’, and ‘C’ sample groups in all pairwise comparisons. Numbers denote total amount of DEGs. The amounts of DEGs found up-regulated in group ‘C’ versus ‘B’ (‘C-B’), or in group ‘C’ vs. ‘A’ (‘C-A’), or in group ‘B’ vs. ‘A’ (‘B-A’) are shown in red color (down-regulated DEGs are shown in blue, respectively). **(E)** Venn diagram shows the amount of specific and shared DEGs.

To search for the biologically relevant transcriptomic alterations that may reflect the formation of distinct immune and stromal TME-related molecular profiles at initial stages of CeCa progression, RNA-Seq was carried out with the use of a panel of fresh tissue specimens that comprised HPV(+) CINs, invasive squamous cell carcinoma at IA1-IIB stages (the group was assigned as “CR”), and normal epithelium from healthy controls. Unsupervised hierarchical clustering of the top 1000 most variable genes yielded a heatmap with two main clusters that overall matched the expected separation among CIN and CR groups (**Figure 1A**; **Supplementary Table S2**; PCA additionally confirmed a clear distinction among the two states, non-invasive and invasive, **Supplementary Figure S2**). However, it turned out that the CR group itself can be further subdivided into two sub-clusters that were designated as ‘A’ and ‘B’. Therefore, a transcriptomic comparison was next performed between the three groups of specimens identified on the basis of similarity of their molecular profiles: group ‘A1-A5’ consisted of microinvasive and invasive CeCa only, group ‘B1-B4’ included CeCa and also one carcinoma *in situ* case, while group ‘С1-С7’ was considered as a comparison group, since it comprised most part of pre-invasive CIN cases. K-means clustering (k = 3) confirmed distribution of groups ‘A’, ‘B’, and ‘C’ between the three clusters (I-III) of coordinately expressed genes (**Figures 1B, C**). GO pathway enrichment was performed on the identified patterns: Cluster I was enriched with epithelial differentiation processes, Cluster II was functionally related to the immune response and inflammatory processes, Cluster III genes shared functions in the extracellular matrix (ЕСМ) associated processes, such as cell adhesion and motility, regulation of cellular morphology, as well as angiogenesis. Significantly, cluster II also turned out to contain genes implicated in maintenance of DNA/chromatin organization and DNA damage response (**Supplementary Table S3**).

Then we identified the genes significantly differentially expressed between the groups of co-clustering samples with a threshold of p-adj. < 0.1 and FC > 2: 809 down- and 552 upregulated DEGs were found in ‘A’ versus ‘C’ comparison, and 679 down- and 217 upregulated DEGs were found in ‘B’ versus ‘C’ comparison (**Figure 1D**), showing a trend of a higher ratio of down-regulated over up-regulated genes in invasive tumor groups. ‘A’ versus ‘B’ comparison yielded 434 down- and 488 upregulated DEG; no significant difference in the level of HPV-derived early gene (E) transcripts was detected between ‘A’ and ‘B’ (**Supplementary Figure S3**). As seen from Venn diagram comparisons (**Figure 1E**), the majority of DEGs were non-overlapping and, besides, these genes were found to be enriched in different GO functions (see **Supplementary Figure S4**). This allows to propose that the early-stage A and B tumors may utilize different mechanisms to sustain progression and may thus constitute different phenotypes. In light of this assumption, we analyzed the range of signaling pathways with the use of GAGE approach.

**Figure 2:**
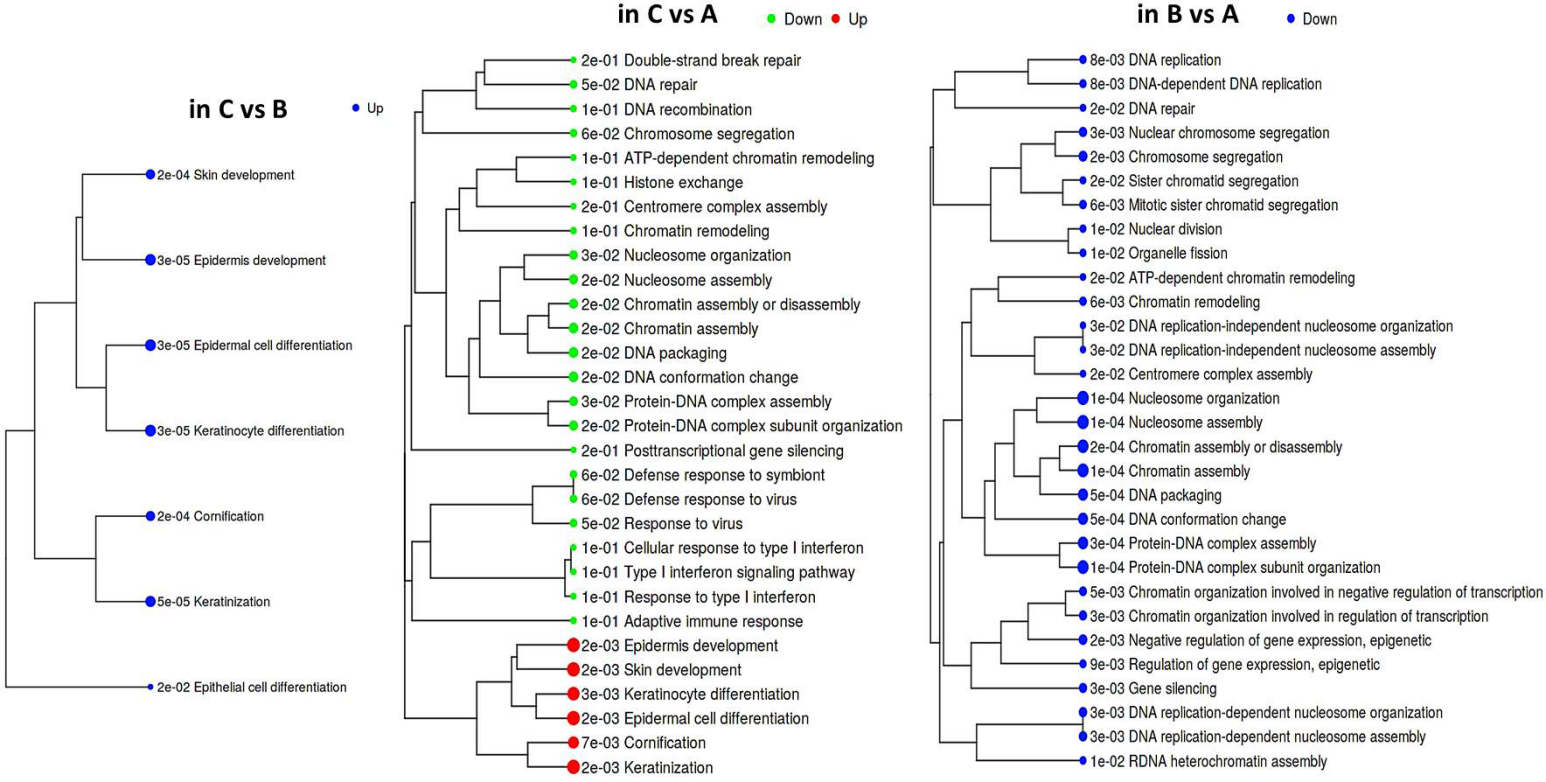
Hierarchical tree of GAGE results for groups ‘А’, ‘В’, and ‘С’ pairwise comparisons. Top 30 pathways were selected with pathway significance cutoff 0.2. The size of dots at the end of branches corresponds to adjusted p-values printed in front of the pathways. Pathways sharing more genes are grouped together (the enrichment statistics and the lists of genes in each pathway are shown in a **Supplementary File 1**).

According to GAGE, repression of the epithelial differentiation program was a common feature distinguishing both groups ‘A’ and ‘B’ from ‘C’ (**Figure 2**; **Supplementary File 1**). Besides, it emerged that group ‘A’ datasets displayed higher prevalence of DNA/chromatin- and immunity-related (including interferon-dependent) gene sets. Regarding chromatin-associated processes, GAGE revealed these include not only spatial chromatin organization per se, but activation of chromatin remodeling and epigenetic regulation (such as post-transcriptional silencing) as well. When searching for the differences between ‘A’ and ‘B’ phenotypes, we got enrichment results for only the gene sets down-regulated in ‘B’ (all of them related to chromatin functioning and its negative regulation), which suggests that a phenotype ‘B’ development is apparently far less associated with epigenetic silencing and chromatin remodeling in general.

Induction of the immune gene sets in group ‘A’ datasets attracts interest, since it contrasts with the known ability of HPV to abrogate immune-defensive, anti-viral, and inflammatory mechanisms. On the other hand, it is well established that chronic antigen stimulation can turn on inhibitory immune checkpoint mechanisms and lead to immune exhaustion/suppression, therefore we looked at the expression profile of individual genes recognized as immune checkpoints (**Supplementary Figure S5**). Indeed, an immune-active group ‘A’ displayed enhanced expression of several modulators, such as BTN3A1/2 co-stimulator/co-inhibitor, a T cell-exhaustion marker SLAMF7, and TIGIT. An increasing trend was also observable for PD-L2 expression. Unlike ‘A’, group ‘B’ displayed significant elevation of an immunosuppressive HMGB1 gene and CD73 nucleotidase, but reduced GZMB expression. These differences in the expression profiles of immune checkpoints offer potentially diverse scenarios for an immunosuppressive microenvironment formation.

To show possible relations between the observed gene expression changes and CeCa phenotypes at initial progression stages, a search for gene co-expression networks was conducted. WGCNA resulted in 10 different modules of the highly correlated genes (**Supplementary Figure S6**). Subsequent GO enrichment analysis pointed out that the mechanisms guiding morphogenetic programs, including angiogenesis, cell adhesion and migration, might be functionally important to the formation of CeCa phenotypic traits at early disease stages and tightly coupled with chromatin maintenance and remodeling (**Supplementary Table S4**), while immune effector pathways appeared to be associated with epithelial differentiation processes.

### 3.2 Identification of differentially expressed chromosome regions (a genome position-related analysis of transcriptomic alterations)

**Figure 3:**
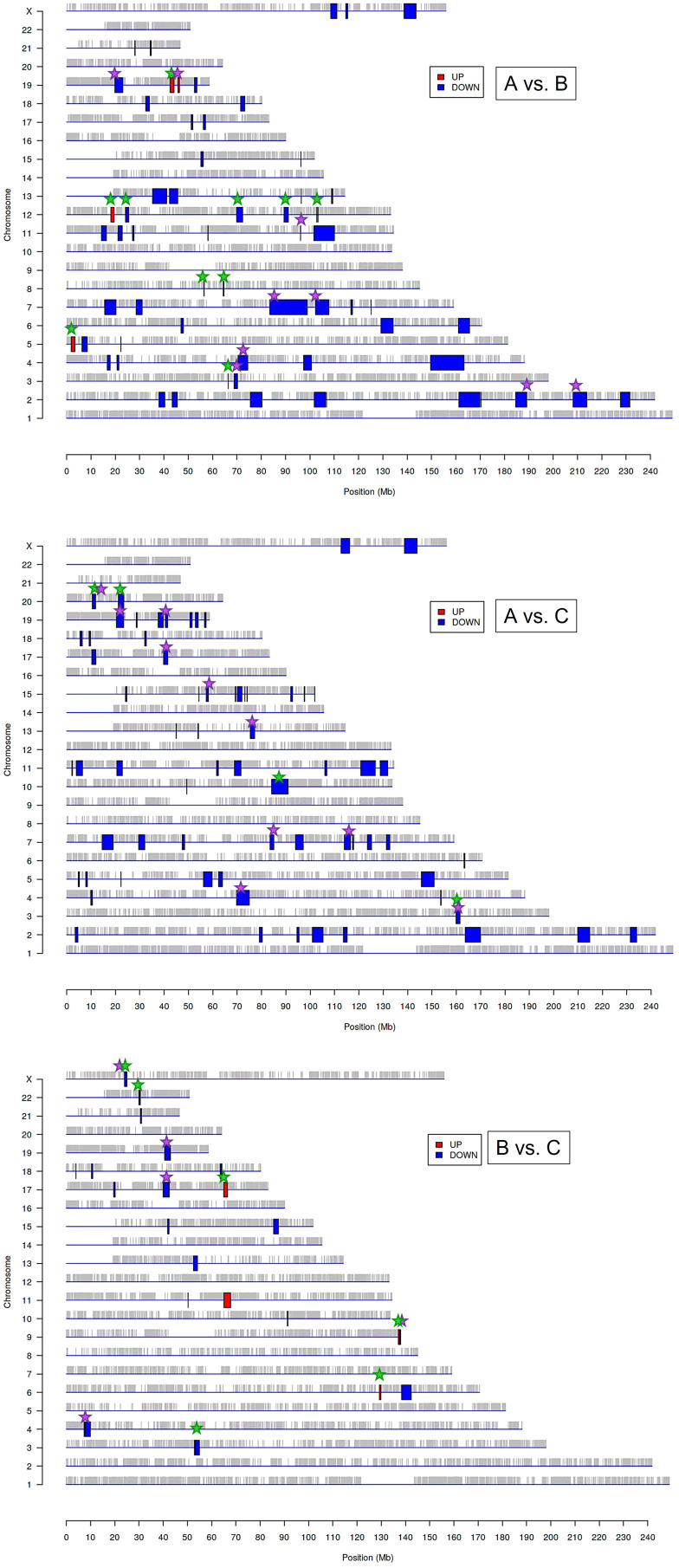
Significantly enriched genomic regions among the DEGs identified by PREDA algorithm in sample groups ‘A, ‘B’, and ‘C’. Blue boxes represent down-regulated regions and red boxes are up-regulated genomic regions. Green starlets indicate group-specific differently expressed regions, and violet starlets designate loci showing overlap with regions, known to contain HPV integration sites described previously in literature (35, 37).

The above results suggest that the chromatin structure-associated processes may play a non-negligible role in diversifying the molecular portrait of CeCa prompting an assumption that not only signaling pathway-level understanding of transcriptomic differences is important, but the genome positional (chromosomal) level also makes sense. It is known that variations in the local structural arrangement of different chromosome regions may influence the functional control mechanisms of genome utilization, or expression; genes co-localized and co-expressed can be potentially co-regulated (34). Furthermore, when dealing with HPV-dependent cancer types, the proximity effect of putative HPV-integration sites should be taken into account (35). To discern chromosomal patterns of highly or weakly expressed genomic regions specific for groups ‘A’ and ‘B’ in relation to ‘C’, we chose to apply PREDA package (34). Significant genomic regions identified by intergroup comparison are graphically outlined in **Figure 3** (information on precise position and gene content is summarized in **Supplementary File 2**). From visual inspection of the resulting chromosome maps it can be inferred that down-regulated DEGs tend to group into relatively extended blocks, while up-regulated genes were more scattered throughout the genome. Besides, of all the DEGs found between groups ‘A’ and ‘C’, only down-regulated genes showed significant association with any of genome regions. Then we examined, which of the differently expressed genomic loci were common for paired group comparisons and which were group-specific (the latter marked by green starlets in **Figure 3**). It indeed emerged that tumor groups ‘A’ and ‘B’ differed from each other and from group ‘C’ by not only certain genome loci, but even different chromosomes appeared to be involved. This is particularly true for up-regulated regions: all the loci up-regulated in ‘B’ versus ‘C’ comparison belonged to other chromosomes than those revealed in ‘A’ versus ‘B’ comparison.

In structural and functional sense, the identified chromosome regions (those being down-regulated, first of all) demonstrated a worth-of-interest picture: first, their gene content showed that many of these regions overlap with tandemly arranged gene families; second, most of these gene families shared common functions in the innate immune response, inflammation, cell death, invasion, and cell identity (**Supplementary File 2**). For example, interleukin (2q11-q12), chemokine (4q13), and siglec gene clusters were found to distinguish group ‘A’ from both ‘В’ and ‘С’. Several other group ‘A’-distinguishing gene families were functionally linked by their role in antiviral response and cytosolic DNA sensing: these are type I/III interferon-inducible IFIT family genes (10q23) and TRIM gene cluster (11p15), as well as IFNΛ cluster (19q13). Furthermore, group ‘A’-specific genome regions contain collections of genes implicated in inflammation and various forms of inflammatory cell death: these are GSDM (gasdermin) cluster involved in pyroptosis (17q21) and group I caspases, namely pro-inflammatory caspases-1, -4, -5, and -12, arranged on chromosome 11q22–23. CARD cluster of caspase inhibitors and cIAP1/2 (BIRC2/3) locus, as well as YAP1 (a transcriptional co-repressor of apoptotic genes), were also found co-localized with this region.

Several chromosome regions specifically expressed in either ‘A’ or ‘B’ groups showed functional linkage to invasion, as they contained clustered protease gene families or their inhibitors, or adhesion molecules: for example, SPINK cluster (5q32) of serine protease inhibitors and KLK locus (19q13) of kallikrein-like peptidases, as well as the cluster of matrix metalloproteinases (ММР, 11q22), were found associated with the group ‘A’. Several differently expressed chromosomal blocks can be considered as determinants of epithelial cell identity, for example, group ‘A’ showed relationship with the KERA, LUM, DCN, and EPYC linkage group (12q21) and with EPGN-EREG-AREG cluster (4q13) of EGF-family members. Group ‘B’ was distinguished by the engagement of CEACAM gene cluster in 19q13 region, as well as squamous cell carcinoma antigen (SCCA) locus (18q21) containing two protease inhibitor and apoptotic inhibitor genes, SERPINB3 (SCCA1) and SERPINB4 (SCCA2). Furthermore, both invasive cancer groups ‘A’ and ‘B’ demonstrated involvement of the pericentromeric 19p13/q13 region containing β-satellite repeats with many embedded ZNF gene family members responsible for transcriptional activation-repression (36) and found oppositely regulated in ‘A’ and ‘B’.

Considering the previously described recurrent HPV-integration sites (hotspots) and in view of the fact that the host cell transcriptional (super-)enhancers, cell-identity genes, and cancer driver genes are often overrepresented in these hotspots (35), we then wondered if the derived genome regions are localized in vicinity of these sites. We visually matched them with HPV-integration hotspots and conventional fragile sites (from (35, 37); violet starlets in **Figure 3**) and found that overlapping regions included super-enhancer-like elements and cancer driver genes such as ERBB4, CASP8, BRCA1, RARA, FGFR3, MET, JAK3, PGR, MYH, PRKCA, POLA1, IDH1, MAP2K1, PPP2R1A, CDK12, SMARCA4, PIK3R2. Somewhat curiously, although chromosome 1 contains a large number of reported integration hotspots, no significantly associated regions were determined in any of intergroup comparisons; chromosomes 8, 14, and 16 likewise appeared unaffected.

### 3.3 Transcriptome-based analysis of cell population composition

**Figure 4:**
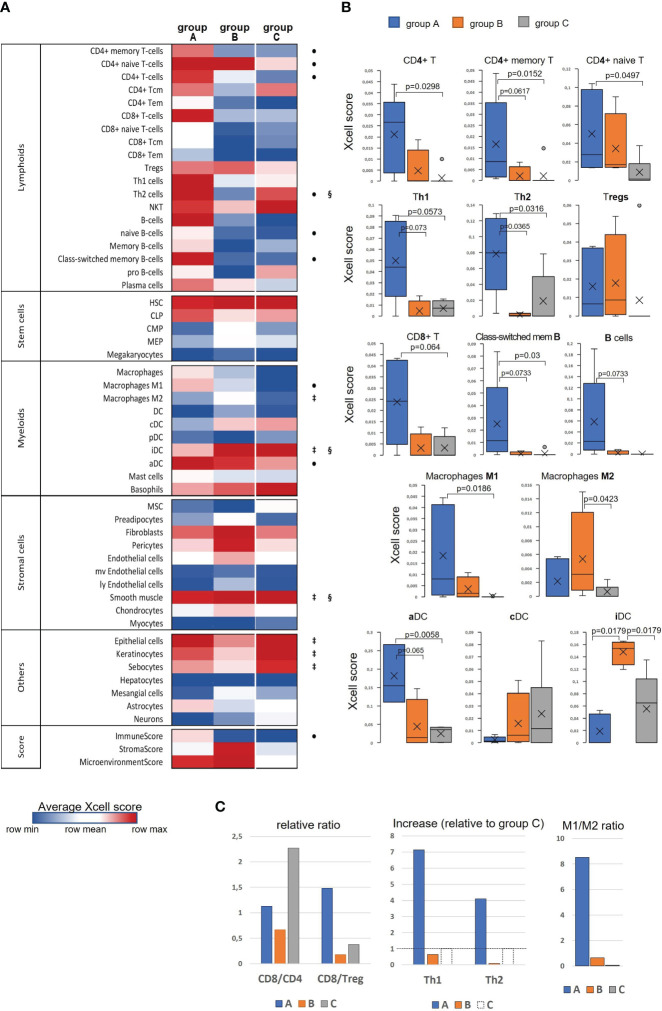
Evaluation of differences in the cell type composition of cervical tissue samples in accordance with the groups they were clustered in ‘A’, ‘B’, or ‘C’: **(A)** Heatmap of xCell enrichment scores. Tem, T effector memory; Tcm, T central memory; CLP, common lymphoid progenitor; CMP, common myeloid progenitor; HSC, hematopoietic stem cell; GMP, granulocyte-macrophage progenitor; MEP, megakaryocyte-erythroid progenitor; MPP, multipotent progenitor; MSC, mesenchymal stem cell; DC, dendritic cell (aDC, activated DC; iDC, immature DC; cDC, conventional DC; pDC, plasmacytoid DC); mv, microvascular; ly, lymphatic. Of all the range of analyzed cell types, MPP, Erythrocytes, Eosinophils, and Osteoblasts were recovered in none of the datasets; regarding the abundance of Tγδ cells, NK cells, Platelets, and Monocytes, a non-zero result was retrieved in only one of 16 samples; as to GMP, Neutrophils, Adipocytes, and Melanocytes – in only two samples, and for that reason these populations of are not included. The significant intergroup differences (p<0.05, Wilcoxon-Mann Whitney U-test) are marked by symbols: • significant difference between ‘A’ and ‘C’, ^‡^, between ‘B’ and ‘C’, ^§^, between ‘A’ and ‘B’; **(B)** Boxplots showing average scores for some most noticeable changes in amounts of infiltrating lymphocytes and antigen-presenting cells; **(C)** Histograms showing several meaningful ratios calculated from the mean-group xCell scores and reflecting the cellular immunity status; group ‘C’ Th1 and Th2 cell counts were taken as 100% (shown by a dashed line) to quantify the relative increase in groups ‘A’ and ‘B’.

Taking into account the finding of an immunologically more active phenotype in a series of tumor samples and detection of stromal TME remodeling features, we decided to examine if these differences in the gene expression and signaling patterns could be manifested in altering cellular composition of the tumors, using xCell algorithm (33). xCell performs cell type enrichment analysis from bulk gene expression profiles for 64 immune and stroma cell types allowing for digital dissection of TME. This is a gene signatures-based method learned from transcriptomes of thousands of pure cell types from various sources and validated using extensive *in-silico* simulations and immunophenotyping experiments (38). Its algorithm represents an alternative to deconvolution approach and enables separation between closely related cell types for cross-sample analysis. Heatmap in **Figure 4A** generated by the use of mean group values summarizes all cell type inferences (some notable parameters of the immune infiltration enrichments are shown in more detail as boxplots in **Figures 4B, C**). Considering lymphoid cell population and, foremost, infiltrating Т cells, substantial differences between the sample groups were derived for the CD4+ Т subset, with its score showing a significant increase in group ‘A’. Although the counts of CD4+ naïve T cells were comparably elevated in both ‘A’ and ‘B’ groups relative to ‘C’, the CD4+ memory T-cell score appeared to be increased only in ‘A’ group suggesting that not only CD4+ cells are increasingly recruited at the tumor site, but in contrast to ‘B’, group ‘A’ tumors exhibited a more efficient immune response development as well. Related to this aspect is an observed distinction in Th1/Th2-differentiation, with ‘A’ and ‘B’ groups showing opposite changes. Specifically, group ‘A’ was distinguished by a sharp increase in enrichment scores of both Th1- and Th2-gene sets (**Figure 4B**), this increase being higher for the Th1 subset (**Figure 4C**). An inverse ratio was seen in ‘B’ tumors: while Th1-cell score almost didn’t alter, that of Th2 cells was dramatically decreased (as compared to group ‘C’, **Figure 4C**). Interestingly, the amount of regulatory T cells (Tregs) showed a clear and comparable trend to an increase in both ‘A’ and ‘B’ groups in relation to ‘C’ pointing at a developing immunosuppression in both cases.

A different landscape of alterations was observed for CD8+ T-cell population: unlike group ‘A’ that displayed an abruptly increased signal from the total CD8+ cell population and its naïve CD8+ T-cell subset, group ‘B’ had reduced frequencies of those cell types compared with group ‘C’, which could be accounted for by not only impaired development of a CD8+ T cell-mediated response, but actually a disruption of cytotoxic T-cell recruitment. Averagely, in group ‘C’ samples (i.e., pre-invasive stage) CD8+ Т cells were about twofold more prevalent than CD4+ T cells, whereas “tumorous” sample groups showed a notable decline of the CD8/CD4 ratio: in group ‘A’ the abundance of CD8+ T cells diminished approximately to that of CD4+ cells, while in group ‘B’ this decline was found even more pronounced (CD8/CD4<1; **Figure 4C**), so that one might even speculate about CD8+ T-cell exclusion. Remarkably, group ‘A’ demonstrated an increase (relative to ‘C’) of CD8/Treg ratio to the values averagely higher than 1; in the meanwhile, this ratio fell to negligibly low levels in group ‘B’ tumors, corresponding to unfavorable settings. Regarding В lymphocytes, group ‘A’ also showed a conspicuous enrichment across the B cell-differentiation lineage (including naïve B cells, plasma cells and memory В cells), so that, according to xCell scores, a В-cell population quantitatively dominated as compared with T cells. A significant increase in the level of class-switched В cells in group ‘A’ may indicate that in these CR-samples the recruited B cells become actively engaged with a specific antibody-mediated response. No such changes were noted in group ‘B’, which could be interpreted as the state of impaired activation of a В cell-mediated response and suppressed formation of the В-cell memory.

The most demonstrative and specific differences between groups ‘A’ and ‘B’ versus ‘C’ were those in the abundances of antigen-presenting cells – dendritic cells (DC) and macrophages (M), and particularly their different functional or polarization states. As seen in the heatmap, immature DC (iDC) constituted a substantial proportion of conventional DC (cDC) in the pre-invasive group ‘C’ (**Figure 4A**). In group ‘A’ the percentage of activated DC (aDC) greatly increased, while that of iDC cells decreased. Conversely, in group ‘B’ the frequencies of iDC cells were significantly elevated as compared with both groups ‘A’ and ‘C’. As to М1/М2-differentiation, group ‘A’ displayed an apparent М1-polarity, whereas group ‘B’ showed a significantly increased percentage of М2-macrophages, so that they became prevailing over М1 (**Figure 4C**).

Among non-hematogenous lineages, epithelial cells were expectedly enriched mirroring squamous carcinoma tumor type (**Figure 4A**, **Supplementary Figure S7**). A decline in the amount of keratinocytes (along with sebocytes as a variant of terminal differentiation state) in groups ‘A’ and ‘B’ versus ‘C’ reaffirms aggravation of de-differentiation and epithelial-to-mesenchymal transition during the onset of invasive growth. However, it should be noted that the abundances of the entire epithelial cell family gene sets decreased most prominently in group ‘B’, whilst group ‘A’ corresponded to a more differentiated phenotype. Of stromal cell populations, fibroblasts and smooth muscle cells were the most abundant across all the samples, this stromal compartment contributing much more to group ‘B’. Referring to the above-described “pro-angiogenic” cluster, we inspected the distribution of relevant cell types: indeed, the whole range of cell types implicated in the microvasculature formation (not only endothelial cells, but such specialized populations as pericytes, lymphoendothelial cells, hemopoietic precursor cells, a mesangium-related subset as well) were present at higher frequencies in group ‘B’, with no significant difference of those between groups ‘A’ and ‘C’. Addressing the combined Xcell-scores, namely ImmunoScore, StromalScore, and MicroenvironmentScore, it can be inferred that the TME impact was detectably higher in both ‘A’ and ‘B’ cancerous groups than that of ‘C’, but it is only group ‘A’ that demonstrated a significantly increased influence from immune infiltration, while group ‘B’ conversely exhibited an expanded role of tumor stroma. Given that ‘A’ and ‘B’ cancer samples comprised mainly the earliest invasive stages, these groups can be viewed as not only different immunophenotypes, but likewise as different scenarios of transition from intraepithelial growth toward active invasion. Conditions to escape immune surveillance, essential for invasive progression, are obviously to form in both tumor groups ‘A’ and ‘B’, but may originate from different sources.

## 4 Discussion

In the present study, main interest was focused on the diversity of transcriptomic profiles of consecutive stages of CeCa initial progression, primarily pre-invasive and early-stage invasive carcinoma. On the one hand, these stages represent a continuity, while on the other hand, they are demarcated by an induction of invasion. We revealed gene expression patterns showing distinctive functional enrichment and potentially representing different molecular phenotypes. Several previous studies dealing with more advanced CeCa stages also reported on the existence of consistent transcriptomic patterns; for instance, Li et al. identified four distinct CeCa phenotypes (‘hypoxia’, ‘proliferation’, ‘differentiation’, and ‘immunoactive’) (39). Lu et al. described two expression patterns, one of which showed a strong immune response, mesenchymal features, and epigenetic silencing, while another exhibited elevated expression of genes involved in keratinization, biological oxidation, and Wnt signaling (40). Li et al. distinguished two CeCa subtypes, one of which was defined as an immune-enriched subtype, while another was found to be enriched in signal conduction associated with angiogenesis, invasion, migration, and metastasis (41). Lyu et al. reported on immune-active and immune-exhausted sub-classes of CeCa, differing in wound healing, IFN and TGFβ signatures (42). Thorsson et al. categorized CeCa into two (of 6 established) pan-cancer immune phenotypes - C1 (“wound-healing”) and C2 (“IFNγ-dominant”) (6).

From pathway enrichment analysis, we found that early stages of invasive CeCa differed from their non-invasive precursor lesions in marked up-regulation of innate immunity pathways, particularly antiviral mechanisms mediated by DNA-sensors, and involvement of IFN-stimulated, pro-inflammatory, as well as DDR-related genes. This probably implies that overstimulation of protective mechanisms in early periods may be one of the triggers and contributors of invasion; at subsequent stages this could become manifest in the form of an immune-active but chronically inflamed and exhausted molecular subtype (43–45). Relatedness of the DDR-associated processes and “heating-up” the immune TME has also been underscored by some researchers (42, 46–48). As regards cell-type enrichment of the immune and stromal TME, our observations on early invasive CeCa are in agreement with the existence of immune-active and immune-suppressed phenotypes (49–51). However, intriguingly, cancer samples with an immune-active profile expressed relatively less well explored checkpoint markers, such as PDL2, BTN3A, TIGIT, and CD73; their dual stimulatory/inhibitory role in the immune response regulation attracts growing interest in view of their diagnostic/therapeutic potential. Detection of these checkpoint molecules could be accounted for by their engagement in the process of TME editing particularly at early stages of invasive progression. PREDA also showed that the discriminative features for early invasive CeCa subsets consisted not only in a suite of particular genes and pathways, but also the genomic loci involved. The fact that the gene loci with downregulated expression tended to be arranged as relatively extended blocks and encompassed a number of anti-oncogenic genes, along with the observed enrichment of chromatin-associated processes and transcriptional regulation, allows supposing virus-induced epigenetic silencing be one of the underlying mechanisms.

It is worth of noting that to date relatively few studies addressed early CeCa stages in the context of phenotypic diversity and the roles of inflammatory and innate antiviral pathways in the transition from intraepithelial development toward invasive expansion (26, 52, 53). One such study is that of Øvestad et al., who applied targeted RNA-Seq across a set of immune-related genes to normal cervical epithelium and CIN3 with the aim to find markers associated with the risk of CIN3 progression (54). Another study compared expression of the immune-related gene set for two CIN3 outcomes, regressing vs. persistent, to elucidate potential drivers of CIN3 progression to invasive carcinoma (55). Wang et al. identified ‘immune-hot’ and ‘immune-warm’ phenotypes both in high-grade intraepithelial lesions and cancers, with both these phenotypes showing features of immunosuppression and pro-inflammation (56). Based on their findings, Li et al. proposed that, while playing a protective function in precancerous lesions, the highly expressed immune-related genes appeared to be associated with worse prognosis in tumor tissue (57).

In conclusion, we acknowledge weaknesses of our present study. An apparent limitation is a small sample size and, accordingly, a moderate statistical power of the results that require further validation in larger sample panels. On the other hand, we consider using of an “in-house” sample panel (consisting mostly of preclinical CeCa stages relatively less covered by research in this field) as the strength of the work. Summarizing the observations from RNA-seq we can infer that early in CeCa progression the immunologic constituent likely plays a key role in orchestrating its traits (such as capacity of promoting invasion and angiogenesis). The results reassure about the rationale for continuing investigation and complementing it with other levels of biologic information.

## Data availability statement

The datasets presented in this study can be found in online repositories. The names of the repository/repositories and accession number(s) can be found below: Gene Expression Omnibus (GEO) with accession ID GSE223804.

## Ethics statement

The studies involving humans were approved by committee on medical ethics at the Institute of Medicine of Petrozavodsk State University and the Ministry of Healthcare of the Republic of Karelia (protocol No.5, Approval date: 1 Dec 2022). The studies were conducted in accordance with the local legislation and institutional requirements. The participants provided their written informed consent to participate in this study.

## Author contributions

Conceptualization and methodology, OK and PK. Obtaining surgical tissue specimens and clinical data curation, PK. Experimental procedures and data acquisition, OK. Bioinformatics, data interpretation, and visualization, PD. Project administration and funding acquisition, TV. Original draft preparation, OK. Manuscript review and editing, PK and TV. All authors contributed to the article and approved the submitted version.
