# Supplementary material for: Sequencing-based transcriptome analysis reveals diversification of immune response- and angiogenesis-related expression patterns of early-stage cervical carcinoma as compared with high-grade CIN
Source: Front Immunol. 2023 Sep 4;14:1215607. doi: 10.3389/fimmu.2023.1215607 (PMC10507244; doi:10.3389/fimmu.2023.1215607)
Supplement: Supplementary file 1 [file DataSheet_1.pdf]

Supplementary Material

Brief Research Report: Sequencing-based transcriptome analysis reveals diversification of immune response- and angiogenesis-related expression patterns of early-stage cervical carcinoma as compared with high-grade CIN

Olga V. Kurmyshkina, Pavel V. Dobrynin, Pavel I. Kovchur, Tatyana O. Volkova*

*** Correspondence:** Corresponding Author: VolkovaTO@yandex.ru

1 Supplementary Figures

Supplementary Figure S1. Histological patterns of a panel of cervical tissue specimens used for transcriptomic analysis. (**A**) Example images of intraepithelial neoplastic lesions with different degrees of severity. In CIN1, dysplastic alterations are observable in the lower third of squamous stratified epithelium (its basal and parabasal layers); in CIN2, morphological abnormalities span 1/3-1/2 of thickness; in CIN3, they expand through the entire thickness but not involving the most superficial cell layer; in carcinoma *in situ*, all cell layers are affected but the basement membrane remains intact. (**B**) Example images of microcarcinoma of the cervix (FIGO stage IA1) with different depth of stromal invasion (in mm). Tissue sections were stained with hematoxylin and eosin (magnification of ×100).

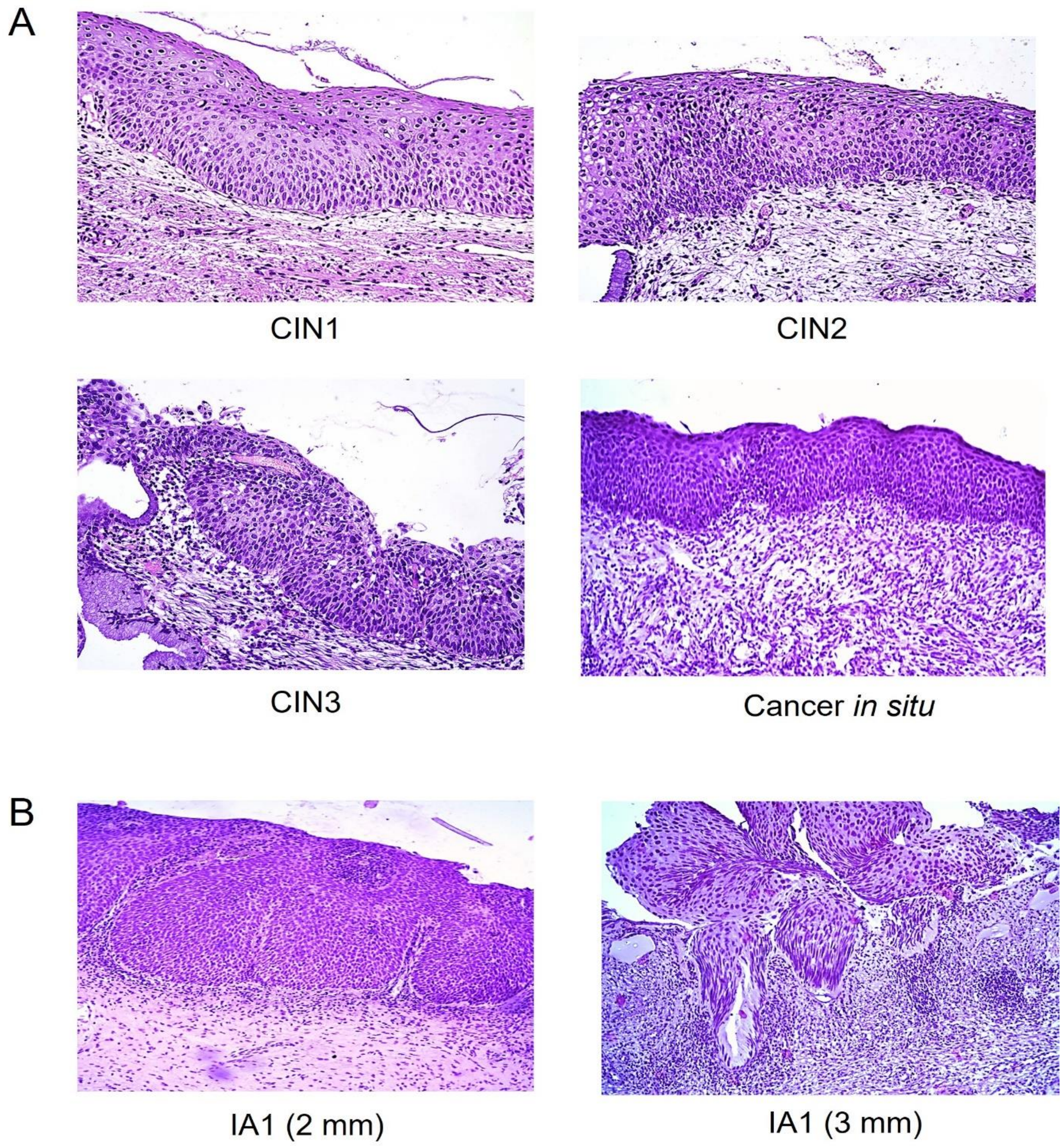

Supplementary Figure S2. Principal component analysis (PCA) of the total RNA expression profiles displayed along the first two principal components (PC), with each point representing a single sample colored according to patient group. The PCA results demonstrated expected grouping among invasive cancer (CR) samples and intraepithelial lesions (CIN). In this plot, the sample CIN_1 is missed out for visual convenience because of large distance between CIN_1 and both CIN and CR samples; when it is included, the first two principal components accounted for >45.0% of the variance in the data.

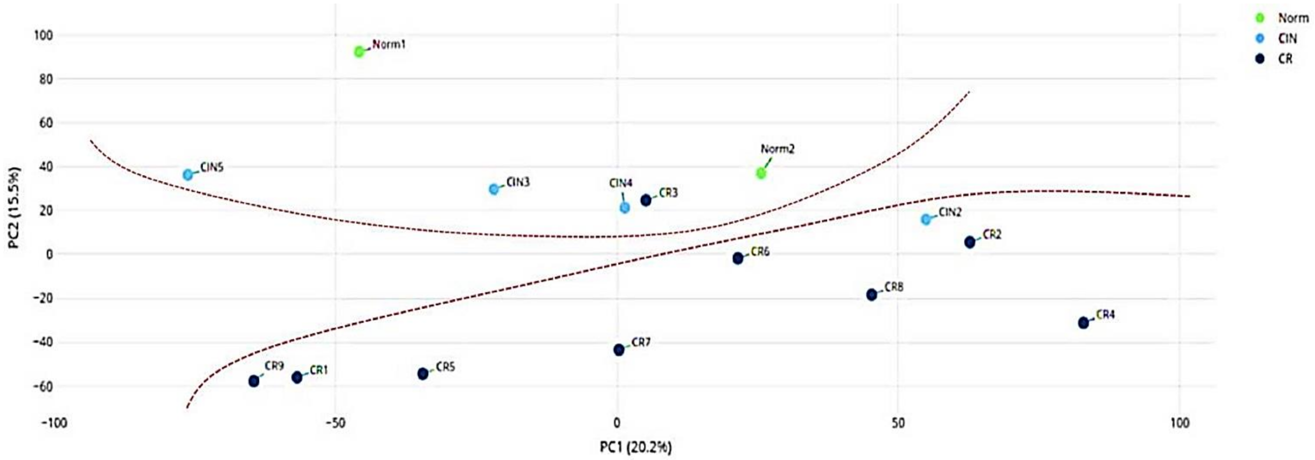

Supplementary Figure S3. HPV16-specific sequencing reads analysis. (**A**) Heatmap depicting sequenced reads counts for early (E1-E7) HPV genes in 'A', 'B', and 'C' groups. (**B**) An example of a mapping summary showing proportion of reads in the CR5 dataset mapped to the HPV16 reference genome (https://pave.niaid.nih.gov/locus_viewer?seq_id=HPV16REF).

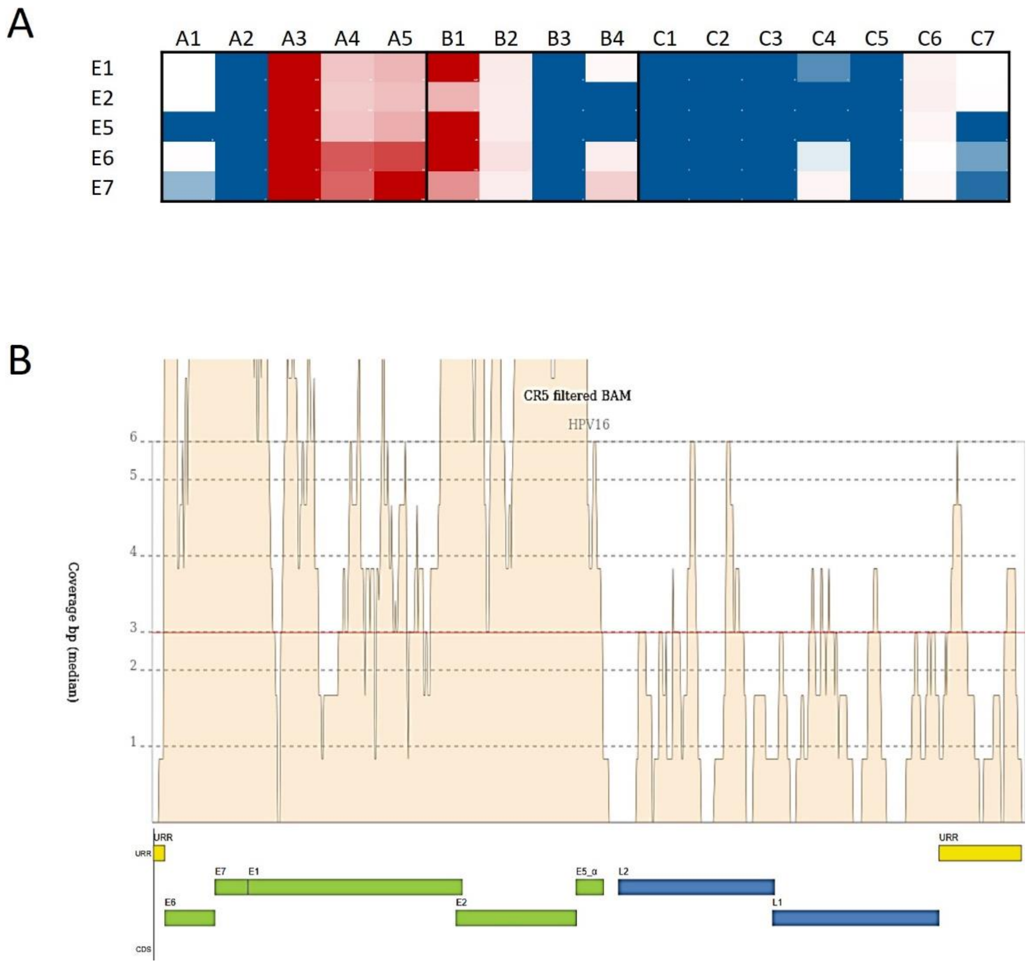

Supplementary Figure S4. GO (Biological processes) enrichment analysis for DEGs found between A', 'B', and 'C' sample groups. In hierarchical trees, the size of red or green or blue dots at the end of branches corresponds to adjusted p-values printed in front of the terms. Terms sharing more genes are grouped together (the lists of genes in each term are available on demand)

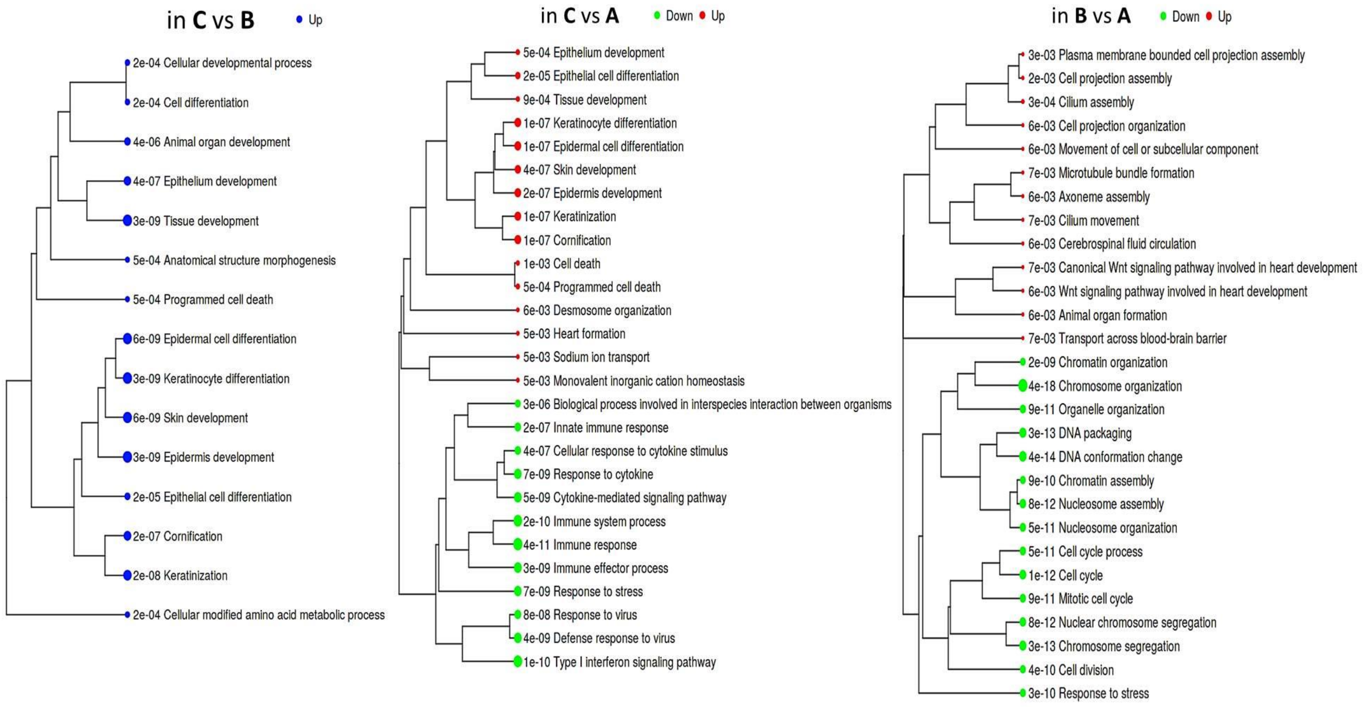

Supplementary Figure S5. Heatmap showing differential expression of common immune modulators or immune checkpoint molecules classified into co-inhibitory/co-stimulatory ligands, receptors, adhesion or antigen-presenting molecules, or others: # significant difference between ‘A’ and ‘B’ sample groups, ● between ‘A’ and ‘C’, § between ‘B’ and ‘C ‘(p-adj. <0.1).

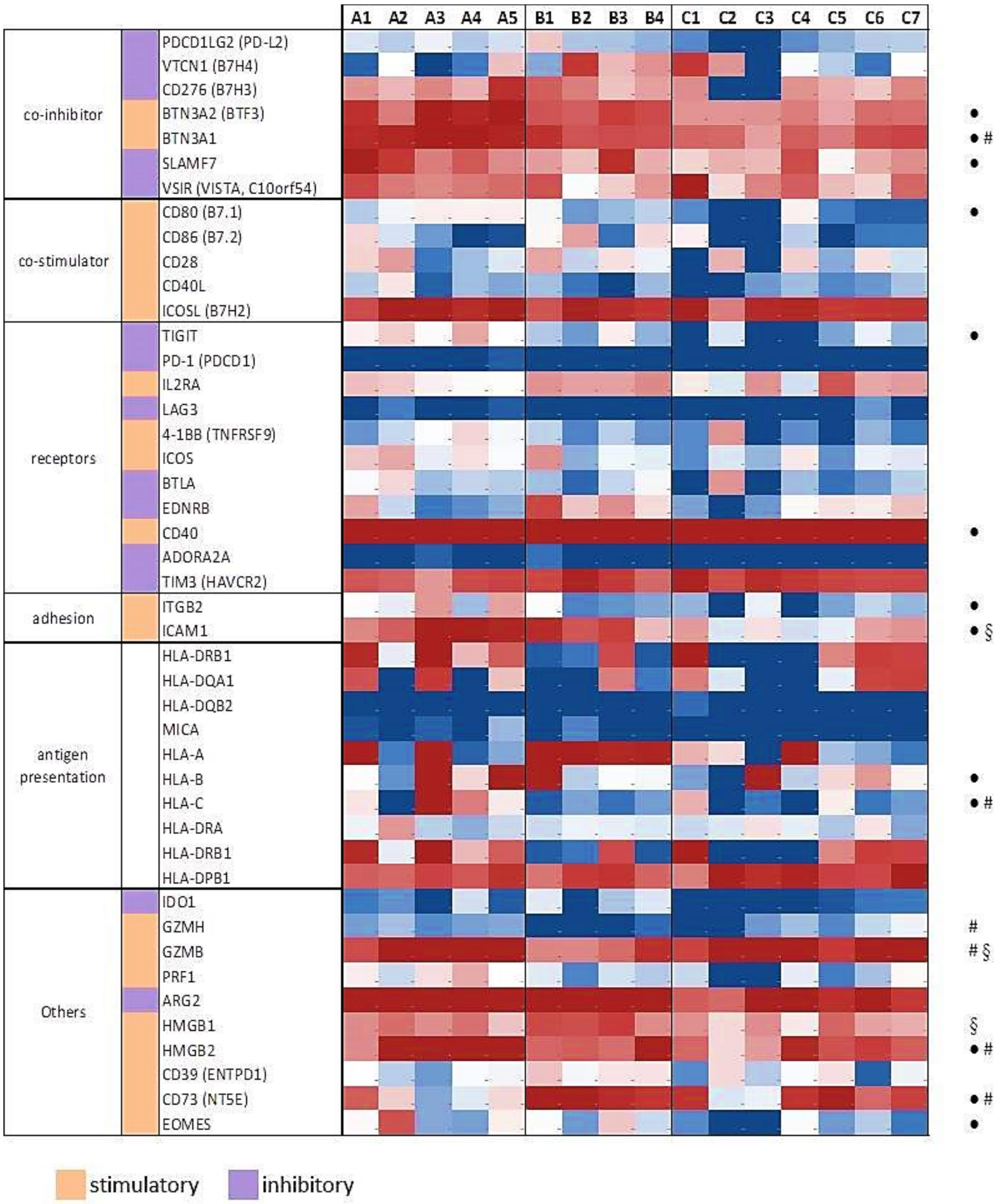

Supplementary Figure S6. Weighted gene co-expression network (WGCNA) analysis across the top1000 most variable genes in the whole sample dataset. In the dendrogram, each branch represents one gene, and every color below represents one co-expression module.

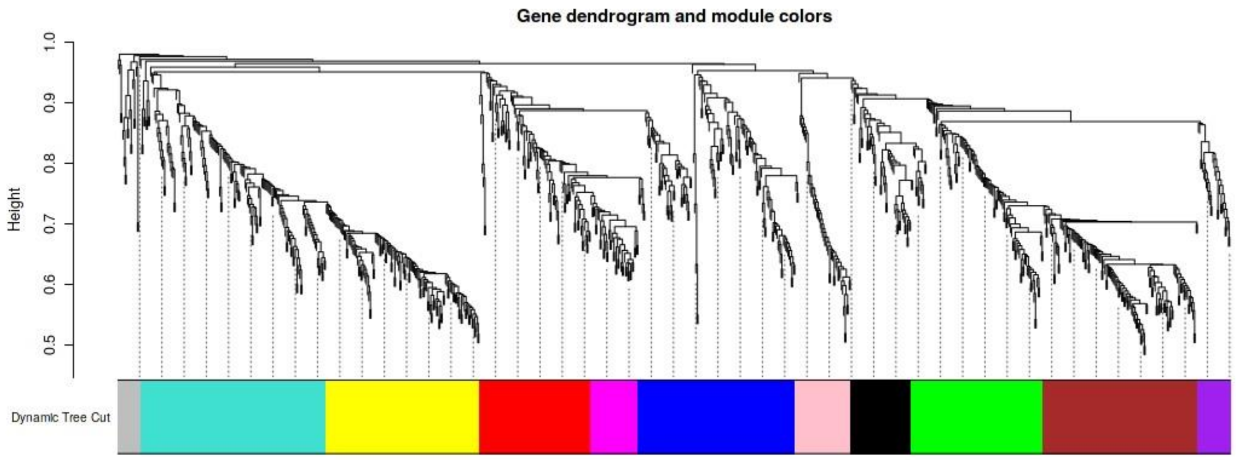

Supplementary Figure S7. Boxplots illustrating differences between sample groups ‘A’, ‘B’, and ‘C’ for the abundances of non-leukocyte types of cells and integrated xCell scores characterizing tumor purity; p-values were drawn by Wilcoxon-Mann Whitney U-test.

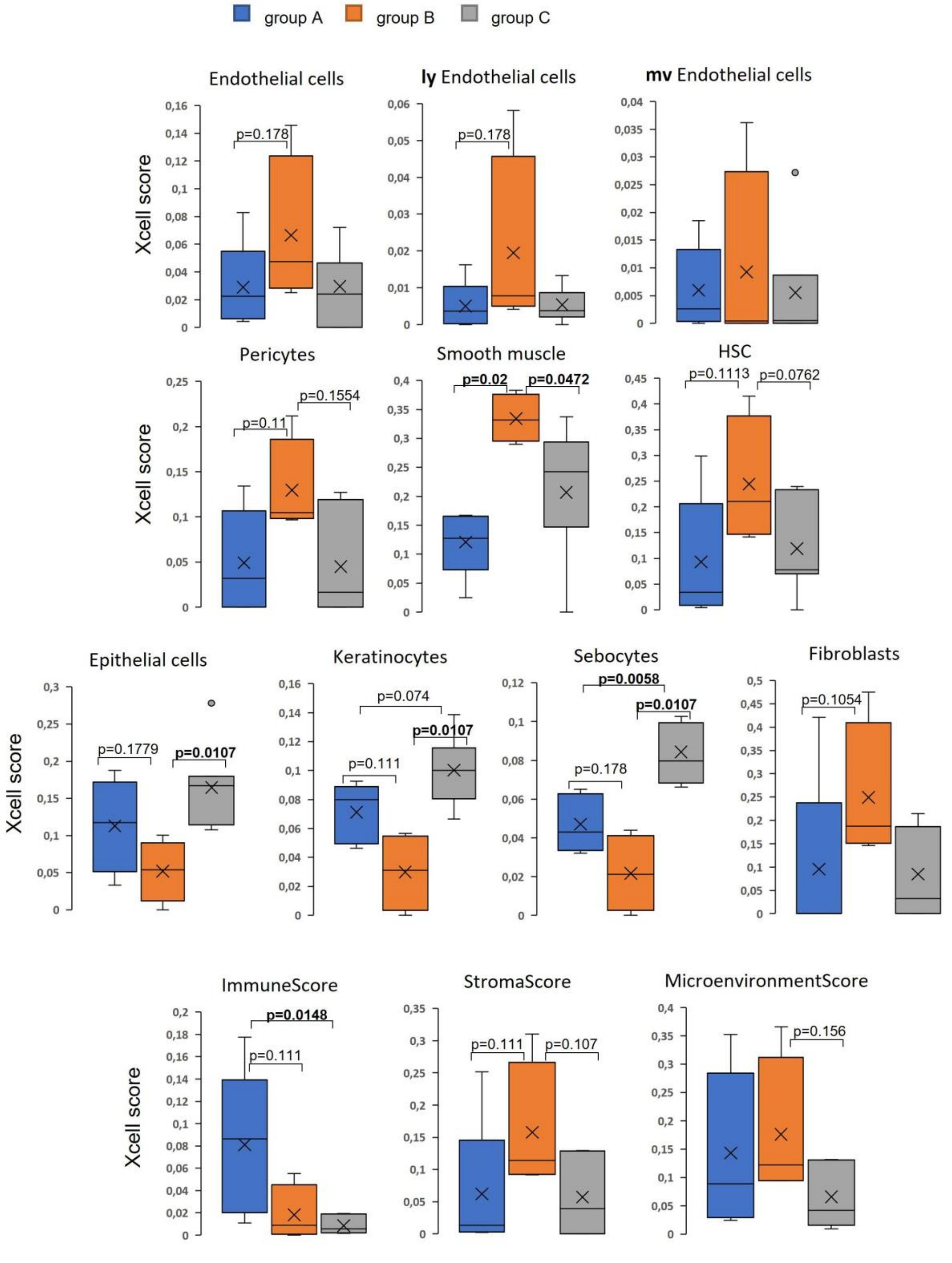

2 Supplementary Tables

Supplementary Table S1: Demographic and clinical characteristics of a set of tissue samples used for transcriptome analysis.

Degree / Stage	Sample ID	Age range	HPV type	HPV load[1]	Comorbidity[2]	Treatment[3]
-	Norm1	25-35		NA	–	–
-	Norm2			NA	–	
CIN3	CIN_1	18-35	HPV16, 31	> 5 lg	arterial hypertension	Surgical (conization)
Cancer in situ (CIS)	CIN_2		HPV16,31,33	> 3 lg	uterine myoma	
CIN2/3	CIN_3		HPV 18, 31, 33	> 3 lg	mycoplasmosis	
Cancer in situ (CIS)	CIN_4		HPV16, 31	> 5 lg	-	
CIN1	CIN_5		HPV 31, 33	< 3 lg	chronic adnexitis	Follow-up
IA1	CR_1	31-40	HPV16, 31	> 5 lg	-	Surgical (conization)
IA1	CR_3		HPV16, 33	> 3 lg	-	
IA1	CR_4		HPV16, 18	> 5 lg	-	
IA1	CR_8		HPV16, 31,33	> 5 lg	arterial hypertension	
IA2	CR_9		HPV16	> 5 lg	-	
IB1	CR_2		HPV16	NA	-	Radical Wertheim hysterectomy / Lymphadenectomy
IB1	CR_5				-	
IB2/IIA1	CR_6	46-55	HPV16	NA	chronic adnexitis	Radical Wertheim hysterectomy / Lymphadenectomy
IIB	CR_7				uterine myoma	

[1] HPV load was measured as lg HPV-DNA/10^5;
[2] 100% of participants were smokers. Number of sexual partners ranged from 3 to 5;
[3] Tissue biopsy uptake was performed prior to any treatment.
NA – not analyzed.

Supplementary Table S2: Unsupervised hierarchical clustering of the top 1000 most variable genes as determined from transcriptome profiles of cervical intraepithelial neoplasia (CIN), invasive cervical carcinoma (CR), and normal epithelial (Norm) samples. Mean normalized log-transformed gene counts are shown.

Supplementary Table S3: Gene Ontology enrichment analysis of gene clusters resulted from k-Means clustering.

Supplementary Table S4: Enriched GO biological processes for each WGCNA module.

Supplementary File 1. Spreadsheets showing Generally Applicable Gene Set enrichment (GAGE) analysis results for groups ‘A’, ‘B’, and ‘C’ pairwise comparisons: top 30 pathways with pathway significance cutoff 0.2 are shown.

Supplementary File 2. Spreadsheets showing PREDA analysis of differentially expressed chromosome regions in sample groups 'A', 'B', and 'C' (pairwise comparisons).
